# Aboriginal Health Workers Promoting Oral Health among Aboriginal and Torres Strait Islander Women during Pregnancy: Development and Pilot Testing of the Grinnin’ Up Mums & Bubs Program

**DOI:** 10.3390/ijerph18189576

**Published:** 2021-09-11

**Authors:** Ariana Kong, Michelle Dickson, Lucie Ramjan, Mariana S. Sousa, Nathan Jones, Ravi Srinivas, Jemma Chao, Joanne Goulding, Ajesh George

**Affiliations:** 1 Centre for Oral Health Outcomes and Research Translation (COHORT), School of Nursing and Midwifery, Western Sydney University, Penrith, NSW 2751, Australia; l.ramjan@westernsydney.edu.au (L.R.); mariana.desouzaesousa@uts.edu.au (M.S.S.); ravi.srinivas@health.nsw.gov.au (R.S.); a.george@westernsydney.edu.au (A.G.); 2 South Western Sydney Local Health District, Ingham Institute for Applied Medical Research, Liverpool, NSW 2170, Australia; 3 Faculty of Medicine and Health, Sydney School of Public Health, University of Sydney, Camperdown, NSW 2006, Australia; michelle.dickson@sydney.edu.au; 4 Translational Health Research Institute, Campbelltown, NSW 2560, Australia; 5 IMPACCT—Improving Palliative, Aged and Chronic Care through Clinical Research and Translation, Faculty of Health, University of Technology Sydney, Broadway, NSW 2007, Australia; 6 Aboriginal Health Unit, South Western Sydney Local Health District, Liverpool, NSW 2170, Australia; nathan.jones3@health.nsw.gov.au; 7 Oral Health Services, South Western Sydney Local Health District, Liverpool, NSW 2170, Australia; 8 Faculty of Medicine and Health, School of Dentistry, University of Sydney, Camperdown, NSW 2006, Australia; 9 The Poche Centre for Indigenous Health, University of Sydney, Camperdown, NSW 2006, Australia; jemma.chao@sydney.edu.au; 10 Primary and Community Services, South Western Sydney Local Health District, Liverpool, NSW 2170, Australia; joanne.goulding@health.nsw.gov.au

**Keywords:** Aboriginal and Torres Strait Islander, pregnancy, oral health, model of care, health promotion, training, participatory action research

## Abstract

Background: this study aimed to develop and pilot test the model of care, Grinnin’ Up Mums & Bubs, to train Aboriginal Health Workers to promote oral health among Aboriginal and Torres Strait Islander pregnant women. Methods: Participatory Action Research was employed to develop the different components of the model (oral health promotion resources, training workshop, and a culturally safe referral pathway to dental services). The model was piloted (pre-post), using an embedded mixed-methods design, to determine the acceptability, satisfaction, and any recommendations made by seven Aboriginal Health Workers at an antenatal service in Western Sydney, Australia. Results: there was a high level of satisfaction with the components of the model of care among the participants, who believed that the model could be integrated into practice. The training showed some improvement in oral health knowledge and confidence. The participants recommended strategies for discussing oral health with Aboriginal and Torres Strait Islander pregnant women, and changes in public health dental policy to ensure that all women would be able to access affordable dental services through the referral pathway. Conclusion: the findings suggest a high level of satisfaction with the model of care among the Aboriginal Health Workers. Further evaluation is needed to confirm the short and long-term impact of the model.

## 1. Introduction

Maintaining good maternal oral health during pregnancy is important for both the mother and the child [1]. Children are more likely to have poorer oral health across the lifespan if the mother also has poor oral health [2,3]. A lack of timely oral health education for mothers may also contribute to oral health or feeding behaviours in children that precipitate dental decay during early childhood [2]. In addition, there is a link between poor maternal oral health during pregnancy and an increased risk of adverse birth outcomes, such as low birth weight or pre-term birth [4]. However, as women have numerous other priorities during pregnancy, oral health tends to be overlooked even in countries with robust healthcare systems, such as Australia. One study found that only about a third of pregnant women living in Western Sydney accessed the dentist in the last six months [5]. Some reasons for the low uptake of dental services among expectant mothers included cost, misconceptions about oral health, and concerns about the safety of dental treatment during pregnancy [5]. Among Indigenous pregnant women globally, further barriers to accessing dental services include a lack of cultural safety, experiences of racism or discrimination, dental-related fears, low self-efficacy, or ineligibility to access public dental services [6].

Australian Indigenous peoples are respectfully known as Aboriginal and Torres Strait Islander peoples, and are acknowledged as the First Peoples and custodians of Australia. Due to a complex interaction of historical, economic, social, cultural, and environmental factors, Aboriginal and Torres Strait Islander Australians experience a greater burden of poor health and disease across their lifespan compared to other Australians [7]. Among Aboriginal and Torres Strait Islander pregnant women, poor oral health can contribute to ongoing economic and health inequalities [8]. Social, political, historical, and economic structural factors can restrict certain oral health behaviours, such as visiting the dentist, for some Australian Aboriginal and Torres Strait Islander pregnant women [6,9,10]. One example of these barriers includes obtaining a Confirmation of Aboriginality, a document issued by an Aboriginal community-controlled organisation that recognises an individual as being Aboriginal and Torres Strait Islander by a community in which the family is known or come from [11]. However, due to past Australian assimilation policies, many Aboriginal and Torres Strait Islander children were removed from their families and respective communities [12]. Such policies have contributed to the complexity in acquiring Confirmation of Aboriginality for many Aboriginal and Torres Strait Islander women. In some instances, individuals may also be able to obtain a Confirmation of Aboriginality where members of their family were born, through family records, or where people recognise the individual as Aboriginal and Torres Strait Islander [11,13,14,15]. Depending on the context, these certificates can support access to culturally safe and affordable services [11] such as dental care at Aboriginal community-controlled health services (ACCHSs) [10]. Other barriers to accessing dental services also include a lack of information and cultural safety among dental services [9,10]. There is a need to capacity build the broader antenatal health workforce and develop health services that provide culturally safe and engaged oral health promotion and support certain oral health behaviours such as accessing the dentist. Previous studies have identified the potential role and need for capacity building of Aboriginal health staff, such as Aboriginal Health Workers and Family Partnership Workers, to promote oral health care among Aboriginal and Torres Strait Islander pregnant women [10,16].

To improve maternal oral health in Australia, the Midwifery Initiated Oral Health (MIOH) model of care was developed to build the capacity of midwives to promote oral healthcare during antenatal appointments, undertake risk assessments and to provide timely dental referrals [17]. The MIOH intervention was found to be effective in increasing the knowledge and confidence of midwives to promote oral health as well as improving maternal oral health and the uptake of dental services during pregnancy [18]. The intervention was also widely acceptable and feasible for all stakeholders [19,20], effective in increasing the uptake to dental services during pregnancy, improved the maternal oral health of pregnant women [17], and was cost effective for health services [21]. The World Health Organization has recently included the MIOH model in their 2021 implementation guidance document as a case study to showcase integration of oral health into primary health care [22]. Although the MIOH model of care was designed to promote oral health care among Australian pregnant women, it was not designed to be culturally specific or relevant for Aboriginal and Torres Strait Islander women. The resources and training for the MIOH model of care were developed without any Aboriginal and Torres Strait Islander community input or engagement, nor involved Aboriginal maternal health staff in the development or delivery of the model. Further, the MIOH training program is widely used by midwives who provide services in mainstream services. However, some Aboriginal and Torres Strait Islander women may choose to access non-mainstream antenatal services for more culturally appropriate care and may subsequently miss out on receiving the MIOH model of care [23]. To create a culturally safe model of care, a more collaborative approach to designing, pilot testing, and implementing the MIOH model needed to be undertaken.

Previous research informed aspects of the MIOH intervention that needed reviewing to promote oral health among Aboriginal and Torres Strait Islander pregnant women and better meet their needs [6,9,10,16]. The aim of this study was to develop and pilot test the intervention, a culturally appropriate model of care with Aboriginal Health Workers to promote oral health during pregnancy; the model of care was named Grinnin’ Up Mums & Bubs. The objectives of this study were to:

1. Develop an evidence-based, culturally appropriate oral health model of care for pregnant women;
2. Pilot test the model of care with the Aboriginal Health Workers to identify the acceptability and satisfaction of the model of care, any improvements in their oral health knowledge and confidence, and future recommendations.

## 2. Materials and Methods

### 2.1. Methodological Approach

This study used Participatory Action Research (PAR) to develop and pilot test the Grinnin’ Up Mums & Bubs model of care. PAR utilises a collaborative approach to define and develop solutions to challenges that hold significance for the participants and community involved in the research [24,25]. PAR challenges the traditional position of researchers and emphasises a shift in roles so that Aboriginal and Torres Strait Islander community members are supported by the research team, ensuring that the research aims, priorities, actions, and outcomes are relevant and reflect the needs of the community [24]. This process situates power with those most affected and encourages self-determination [24].

**Figure 1:**
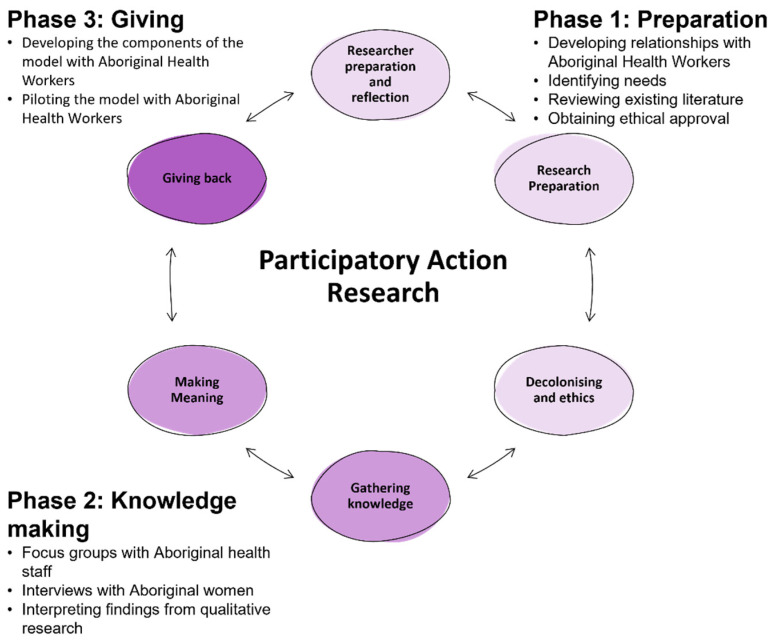
Participatory Action Research framework to guide Phases 1–3.

A PAR framework was adapted from the work of Kovach [26]. This framework involved three iterative phases: Phase (1) Preparation; Phase (2) Knowledge making; and Phase (3) Giving (Figure 1). As with an action research methodology, this was a cyclical rather than a linear process. Preparation (Phase 1) involved developing relationships with the Aboriginal health staff (who were both researchers and participants in the study), identifying the need for the research, conducting and publishing literature reviews [6,16], and obtaining ethical approval. Knowledge making (Phase 2) involved gathering and understanding the knowledge learned through focus groups with Aboriginal health staff [10] and interviews with Aboriginal women [9] to discuss needs and promoting oral health during pregnancy. The knowledge learned from Phases 1 and 2 informed Phase 3, this present study, which aims to give back through the development and piloted testing of a model of care with the Aboriginal Health Workers.

### 2.2. Design

The pilot in this study was evaluated using a mixed-methods concurrent embedded design. This model was piloted with the Aboriginal Health Workers using a qualitative approach, supplemented by quantitative data. In this embedded mixed-methods design, the qualitative component provided valuable and insightful feedback on the acceptability and feasibility of the model as well as recommendations for the next cycle of PAR. The minor quantitative component aimed to complement the qualitative pilot and determine whether there was an improvement in knowledge and confidence in promoting oral health among the Aboriginal Health Workers, and to measure the satisfaction with specific aspects of the model of care.

### 2.3. Study Context

The need for improved oral health among Aboriginal and Torres Strait Islander pregnant women within the community was identified by Aboriginal health staff (including both Aboriginal Health Workers, Family Partnership Workers, and Aboriginal management staff) who worked in Greater Western Sydney, New South Wales (NSW), Australia, prior to the study. As part of Phase 1, the lead author (AK), a non-Indigenous woman, yarned with the Aboriginal health staff about how the Aboriginal Health Workers could drive the research. Yarning is a way of conversing to exchange Aboriginal and Torres Strait knowledge, ideas, experiences, and stories, and is also used to build trust and respect [27]. Since PAR involves community members who both drive the research and participate in the research, it requires a long-term commitment. In this study, a small group of Aboriginal health staff were closely involved in the research from the development stages to the implementation and pilot of the workshop, a period of three years. Although the group of Aboriginal health staff who were part of the study was small, the close and long-term partnership fostered a rich exploration of the needs and recommendations for developing a culturally safe oral health model of care.

### 2.4. Ethical Considerations

Ethical approval was obtained from the South Western Sydney Local Health District (2019/ETH09963), the Aboriginal Health & Medical Research Council (1438/18) and the Western Sydney University (RH13086) human research ethics committees. Written, informed consent was obtained from all Aboriginal Health Workers who participated in the pilot study.

### 2.5. Developing the Grinnin’ Up Mums & Bubs Model of Care

As part of the planning for the model of care (Phase 1), literature reviews were initially carried out [6,16] to identify current gaps in this area and whether other models of care or programs had been developed. The Aboriginal health staff also suggested that focus groups with other Aboriginal health staff [10], and interviews with Aboriginal and Torres Strait Islander pregnant women and mothers of Aboriginal and Torres Strait Islander children [9], would inform ideas for the model of care. These ideas included developing a suite of oral health promotion resources, a training workbook and a training workshop, and implementation of a culturally safe priority referral pathway to the dental service reinforced through the resources and training.

#### 2.5.1. Oral Health Promotion Resources

An Aboriginal graphic designer needed to be employed to create the artwork for the resources. The Aboriginal health staff identified that the graphic designer should be a woman (as pregnancy was seen as women’s business) and reside locally. The graphic designer who was employed to design the art was identified through Supply Nation, a national directory for verified Aboriginal and Torres Strait Islander businesses in Australia.

The specific resources that would be developed to assist with oral health promotion among clients, including ideas for illustrations, content, colours, and designs, were identified and also workshopped collaboratively by the Aboriginal health staff, the graphic designer, and AK. The resources that were considered to be most useful included a brochure, a fridge magnet, and a whiteboard educational tool that could be used by Aboriginal Health Workers as they worked with Aboriginal and Torres Strait Islander pregnant women and mothers on oral health.

The initial designs for the resources were stepped through an extensive consultation process that sought input, ideas, and feedback from the Aboriginal Action Group, Aboriginal and Torres Strait Islander pregnant women, and mothers and dental and nursing staff employed at ACCHSs across metropolitan and rural NSW. This process resulted in important changes to the resources, including changes to the layout, graphics, the language used, and modifications to the key health messages contained in the resources.

Three key messages were used across the resources to promote oral health during pregnancy:

1. Tooth and gum problems are more likely during pregnancy;
2. Your oral health can affect your baby;
3. Dental treatment is important and safe during pregnancy.

The brochure, whiteboard educational tool and training workbook included a self-administered screening tool, and all resources included options to access the dental service. The validated screening tool has a 94% sensitivity in detecting oral health problems among pregnant women [28]. The brochure and two fridge magnets were designed to be given to clients of the Aboriginal Health Workers, after yarning about the importance of oral health during pregnancy. The brochure contained detailed key messages whereas the fridge magnets included a simple call-to-action message. The double-sided whiteboard adapted the screening tool so that clients could interact with the resource using magnets. Images of these resources can be located at Supplementary File S1.

#### 2.5.2. Training Program and Workbook

The format, content, and delivery method of the training program were informed through earlier research with the Aboriginal Health Workers [10] and the MIOH training program [18]. The training was also shaped through consultation with Aboriginal and Torres Strait Islander researchers (MD, JG, NWB, JA) as well as non-Indigenous researchers (AK, AG, LR, MSS). A training workbook, which summarised the information delivered, was also produced as a point of reference for Aboriginal Health Workers to use after they had completed the training workshop.

#### 2.5.3. Referral Pathway

**Figure 2:**
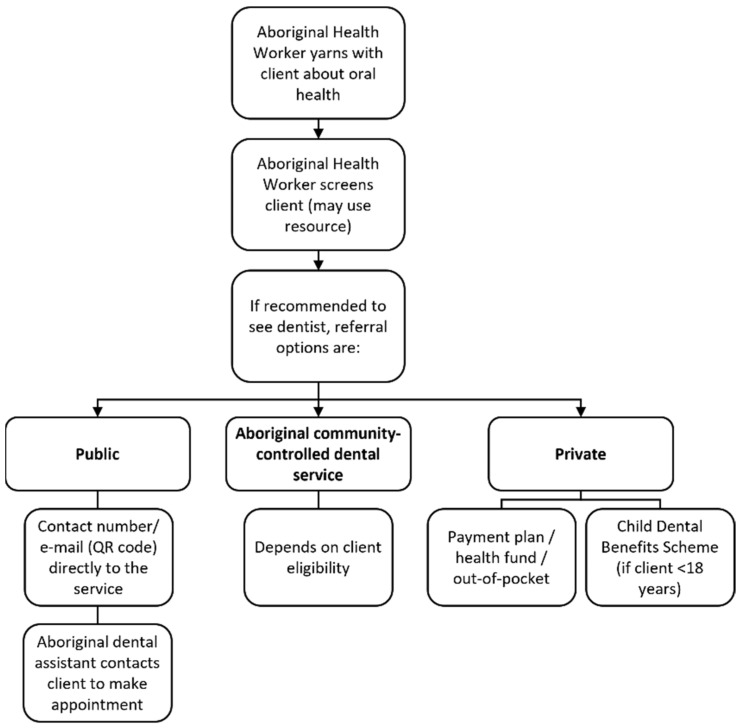
Dental referral pathways for the Grinnin’ Up Mums & Bubs model of care.

Identifying culturally safe and accessible dental service options was a key area of discussion in the earlier phases of the study. The referral pathways implemented were designed to be specific to the community where the study was conducted. There were three main pathways where Aboriginal and Torres Strait Islander pregnant women could access dental services: public dental clinics, ACCHSs, and private dental services. The training provided the Aboriginal Health Workers with information about the different pathways and eligibility requirements of each pathway (see Figure 2).

As this study was conducted in collaboration with a NSW public health service, a strategy to improve cultural safety to access public dental services within public health policy was discussed. According to the existing NSW Health policy, pregnant women who want to book an appointment at a public dental clinic need to telephone a call centre where they are triaged by administration staff to determine eligibility and how soon an appointment needs to be made [29]. The policy stipulates that pregnant woman should not wait more than three months. To improve cultural safety of the local public oral health service, the process for booking dental appointments was adjusted. Either the client (or Aboriginal Health Worker on behalf of the client) could call or e-mail the service directly to leave a name and number. A QR code was also introduced on the fridge magnets so that clients could scan and send an e-mail directly to the dental service to book an appointment. An Aboriginal dental assistant would then call the client back to book an appointment based on whether the client was eligible and the urgency of the client’s oral health needs.

### 2.6. Piloting the Model of Care with Aboriginal Health Workers

#### 2.6.1. Demographics

A total of seven Aboriginal Health Workers participated in the training course and completed the pre-post surveys. The Aboriginal Health Workers were employed by a government public health organisation. Two sites in Greater Western Sydney were originally involved; however, due to the impact of the Coronavirus 2019 (COVID-19) pandemic, the other study site could not participate in the piloting of the intervention. The age of the participants ranged from 26 to 47 years. The highest level of education attained by four of the Aboriginal Health Workers was a certificate or diploma achieved from a vocational education training provider. The other three participants had, or were currently studying towards, a bachelor’s or post-graduate degree. The years of experience working as an Aboriginal Health Worker ranged between seven months to 12 years. Four participants identified as receiving previous education or training regarding oral health care for pregnant women at work in-services.

#### 2.6.2. Recruitment and Data Collection

The Aboriginal Health Workers who participated in the earlier focus groups [10] or were employed at the antenatal service in Western Sydney were invited through e-mail to participate in the study. Recruitment flyers and the participant information sheet were shared with the Aboriginal Health Workers. The Aboriginal Health Workers were given participant information sheets (both online and as a hard copy), which was also verbally discussed with the lead author before providing written consent.

The model of care was piloted with the Aboriginal Health Workers through a pre–post test design. Piloting of the model consisted of delivery of the training workshop and feedback on the resources as well as feedback on the referral pathway using a pre-post questionnaire.

#### 2.6.3. Pre–Post Questionnaire

The pre–post pilot questionnaire (Supplementary File S2) collected qualitative and quantitative data about the training workshop, the oral health promotion resources, and the referral pathway. The baseline pre-questionnaire used questions about antenatal oral health knowledge (Section 1) and confidence (Section 2), in addition to demographic questions. The participants could respond to the knowledge questions by selecting “True”, “False” or “Don’t know”. The confidence questions used a numerical (1–5) Likert Scale ranging from “Not confident at all” (1) to “Completely Confident” (5). The post-training questionnaire included the same knowledge and confidence questions, but also included a feedback component (Section 3) where participants could provide feedback on the relevance, usefulness, and cultural appropriateness of the different components of the model of care. The feedback in Section 3 was provided through a numerical (1–5) Likert Scale ranging from “Strongly disagree” (1) to “Strongly agree” (5) in response to the various statements. In addition, Section 3 included three open-ended questions that were asked at the end of the post-questionnaire:

1. What did you like about the training/model of care?
2. What did you not like about the training/model of care?
3. Do you have any recommendations to improve the training/model of care?

The knowledge and confidence component of the pilot questionnaire was developed from the literature [1,30,31], and included some knowledge and confidence questions used in the MIOH training program for midwives [18], the focus groups with the Aboriginal health staff [10], and from current health policies [29,32]. The questionnaire was piloted with a group of Australian Aboriginal and non-Indigenous academics and researchers, a researcher who previously worked as an Aboriginal Health Worker, and a biostatistician, and changes were made accordingly to ensure readability and cultural appropriateness.

#### 2.6.4. Training Workshop

The training program consisted of a face-to-face workshop delivered by Aboriginal researchers (NWB, JA), a researcher with a dental background (AG), and the lead author (AK). Three training workshops were delivered due to the conflicting schedules of the Aboriginal Health Workers. Depending on the size of the workshop, they were between one to two hours and included a PowerPoint presentation and group discussions. The content comprised of a theoretical component followed by a practical component. The theory included basic tooth and gum anatomy; the relationship between oral health and pregnancy; common problems; guidelines; safety of dental treatment, and good oral health practices. The practical component included a discussion about the need to yarn, screen and refer clients (where appropriate) to the dental service. This component also consisted of discussions about how the oral health promotional resources could be used in the Aboriginal Health Workers’ practice, as well as the appropriate referral pathways and eligibility requirements.

The pre-questionnaire was administered to the Aboriginal Health Workers prior to the training workshops. After the training workshops were delivered to the Aboriginal Health Workers, the follow up post-questionnaire was administered in person. The Aboriginal Health Workers were provided the opportunity by the workshop facilitators to elaborate on the three open ended questions verbally after completing the written questionnaire. All participants consented to have the verbal feedback recorded. The verbal discussions of the three open-ended questions lasted between six and twenty minutes.

### 2.7. Analysis

#### 2.7.1. Qualitative

The qualitative data were transcribed through an external professional transcription service, specialising in academic transcription services. The written qualitative feedback was also included. A conventional content analysis approach, as described by Hsieh and Shannon [33], was used to analyse the data as the participants provided both verbal and written feedback in response to three specific, open-ended questions. As part of the first step, AK read and re-read the transcripts for immersion and inductive category development. AK then highlighted exact words or phrases spoken by the participants that captured key concepts; AK exported these as quotes onto an Excel spreadsheet, and separated the quotes based on which open-ended question they addressed. The quotes were then coded, labelled and categorised based on the ideas shared, which were reviewed by LR for accuracy. AK developed the coding structure by grouping similar codes together to construct meaningful sub-categories. These sub-categories were reviewed and refined together with LR to create broader categories, which were labelled and defined. The codes were then reviewed against the categories. The categories and their definitions were further refined by AK and LR. AK selected specific examples from each category to support the reporting of the data.

#### 2.7.2. Quantitative

Descriptive statistics were used to analyse the quantitative data. To measure the change in oral health knowledge, the pre- and post-questionnaire mean scores for each question were calculated separately and compared. The oral health knowledge score for each question was based on the mean number of correct responses across the group. Changes in oral health confidence, for each question, were measured by calculating the mean scores for both the pre- and post-questionnaires. The scores for the agreement statements in the post-questionnaire feedback, with the various components of the model of care, were calculated as an average out of 5.00. Overall agreement was observed if the score was greater than 4.00 (>80%). The data were analysed using SPSS Statistics for Windows, Version 25 (IBM Corp, Armonk, NY, USA). See Supplementary File S2 for additional details of the quantitative data. In keeping with the mixed-methods concurrent embedded design, the descriptive statistics were integrated into the relevant categories and sub-categories to complement the qualitative data.

## 3. Results

There were three categories that reflected the pilot feedback from participants (Table 1).

### 3.1. Satisfaction with the Model of Care

The participants provided positive feedback on the model of care, and described the components that were useful, relevant, and appropriate.

#### 3.1.1. Satisfaction with the Training: “It Built on My Existing Oral Health Knowledge”

The Aboriginal Health Workers discussed the content of the training, and specifically about how its content was relevant, coherent, and built on the Aboriginal Health Workers’ existing knowledge.

*I think it’s [training] really great. Honestly, the information that you’ve given us isn’t mind blowing. So it’s not like overly technical or anything. It’s just like, oh damn, I didn’t know that which is really good, so it’s easy to understand.*

(Isobelle)

Some Aboriginal Health Workers also provided feedback on the delivery of the training, stating that *“I think it was well-explained”* (Rachel), *“Clear and to the point”* (Anonymous written feedback), and that “*The information was precise and didn’t waffle on”* (Rebecca).

The data from the post-training questionnaire identified that all Aboriginal Health Workers strongly agreed that the content of the training was easy to understand, the length of the training was adequate, relevant to their work, and provided knowledge to use when giving oral health advice to clients (Table 2). All Aboriginal Health Workers were satisfied with the quality of the presenters (100%) and PowerPoint presentation (97%) and found it to be culturally appropriate (97%).

#### 3.1.2. Satisfaction with the Resources: “It’s a Great Visual Prompt”

The participants also reflected on the visual appeal and usefulness of the oral health promotion resources, including the brochure, fridge magnets, the screening tool and the use of a QR code on the resources to make dental referrals. The resources, particularly the brochure, were described as beautiful, bright, colourful, practical, and user-friendly.

*I think the resources are beautiful. They’re nice and bright and user friendly and you don’t feel shame looking at them. You enjoy looking at them.*

(Isobelle)

*I find the QR and with that e-mail [on the fridge magnet]—how it’s literally, pretty much all done, and all you’ve got to do is add your details—name and [number]—so I thought that’s pretty good.*

(Rachel)c

*Appropriate as well as culturally, love the resources. *

(Anonymous written feedback)

The data also corroborated with the Aboriginal Health Workers’ comments about the supporting oral health promotion resources. The post-training questionnaire found that all participants were satisfied with the quality of the resources, and strongly agreed that the oral health promotion resources were culturally appropriate (Table 2).

#### 3.1.3. Satisfaction with the Referral Pathway: “It’s a more Culturally Appropriate Pathway”

All participants agreed that the referral pathways were culturally appropriate (100%) and would be appropriate to use for their clients (97%) (Table 2). The referral pathways to dental services were described as being clear for the Aboriginal Health Workers to use, and that the screening tool supported practice. The comment made by Rebecca was reflected in all the Aboriginal Health Workers’ feedback questionnaires:

*There are also ways to—clear referral pathways for us Health Workers to be able to refer to support information and delivery. And a clear screening tool. So walking with them a little more than just you know—giving them the information and that it’s recommended. When you’re using the screening tool it’s more of a prompt: “Let’s focus on this today.”*

(Rebecca)

### 3.2. Integration of the Model in Practice

The Aboriginal Health Workers considered how the components of the model of care, particularly the training, could be applied in practice and could inform or facilitate change due to an increase in knowledge and confidence levels.

#### 3.2.1. Applying New Knowledge in Practice: “That Information I can then Put into Practice”

Some Aboriginal Health Workers, such as Mia, reflected on how the training had built on their existing knowledge about oral health and pregnancy.

*The information that you’ve provided me today in regards to the health risks of pregnant women—it’s given me reminders about the whole concept of the links between oral health and pregnancy.*

(Mia)

The above comment was evident in the pre–post quantitative data on oral health knowledge. All Aboriginal Health Workers demonstrated an increase in oral health knowledge score. Pre and post scores for each item have been outlined in Table 3. The greatest improvements in knowledge observed were on oral health changes and misconceptions during pregnancy (average 50% improvement). There were also improvements in knowledge about accessing public dental services (39.3%), specific dental treatments that are safe during pregnancy (25.7%), and the link between gum disease and pregnancy outcomes (23.8%).

Many Aboriginal Health Workers went on to relate specific examples where this knowledge could be integrated into their practice. Some described they would change the way that they screened and referred clients to dental services following the training.

*Before I just recommending that everybody see the dentist but obviously now I know that if somebody says ‘yes’ to any of those things [oral health problems] then you obviously have to be a bit more—you have to make that appointment [because] they might be at risk? *

(Rebecca)

#### 3.2.2. Renewed Confidence to Discuss Oral Health: “I Feel more Confident to Answer Questions”

Some participants described feeling more confident to discuss oral health with clients following the training. The Aboriginal Health Workers spoke about how they felt more confident to answer oral health-related questions with clients and reflected that confidence would also be fostered over time with experience.

*I feel more confident to answer questions from now, whereas previously I suppose when it has arisen and they’ve asked me things that I didn’t know I’ve been honest with them and said, I don’t know but let me find out for you.*

(Kim)

*But I think it [resources] will be easy to use, easy to talk about as we get more comfortable talking about it [oral health] as well.*

(Isobelle)

Some participants, such as Samantha and Kim, remarked on how using the resources would help with confidence in discussing oral health as well as taking a systematic approach to giving advice to clients:

*I like how the pamphlet has got that section here where you can ask about what exactly if they’re experiencing any of those…it helps me to not feel worried about forgetting asking something because it’s in there anyway. So you just cover all your bases.*

(Samantha)

*I think with these resources I feel more confident discussing it.*

(Kim)

The pre-post quantitative findings found that all Aboriginal Health Workers were confident to discuss good oral health care with pregnant women, which improved by 17% (Table 4). Following the training, the post-questionnaire found that all participants were confident to ask screening questions to identify if a client needed to see the dentist (20% improvement). All Aboriginal Health Workers were completely confident to refer clients at risk of poor oral health to the dentist (11% improvement).

### 3.3. Further Recommendations

There were no aspects of the model of care that the Aboriginal Health Workers disliked. However, several recommendations around future practice, policy, and the scope to upscale the model were identified. The participants also made some recommendations on how to discuss oral health with clients, particularly if it was seen as a sensitive topic for the client.

#### 3.3.1. Getting the Message out There: “We’ve Got to be Flexible and Meet the Client’s Needs”

Several strategies were recommended by the Aboriginal Health Workers to mitigate the sense of confrontation associated with yarning about oral health. Some reflected on experiences where non-Indigenous nursing and midwifery staff *“aren’t always culturally appropriate”*. (Isobelle)

*Because I’ve had midwives - and the client has just swore and whatever at them because how they’ve come out and said it and pretty much was pretty abrupt and said, you need to book yourself into the dentist because your teeth are very poor and that’s going to do harm to your baby.*

(Rachel)

The need for flexibility in how and when oral health promotion is delivered was discussed by a few Aboriginal Health Workers. The importance of building rapport was stressed, which meant that there needed to be flexibility when initiating a conversation about oral health.

*You build up that rapport and you know that’s going to be an okay conversation to have with someone. we’ve got to be flexible in our approach and meet the client’s needs where they’re at, and it might not be until the fifth or sixth visit.*

(Isobelle)

One of the strategies, suggested by a few Aboriginal Health Workers like Kim, was to take an indirect approach by firstly discussing the oral health needs of the children or family before asking the client.

*So sometimes I’ve had to bypass that and go other ways and talk about the kids that are already in the house with their dental and break it down like that.*

(Kim)

Using the oral health promotion resources to facilitate the conversation was also advised by a few participants. Some suggested that the whiteboard educational tool could be given to a client before an appointment to initiate a conversation about oral health:

*I think for me it would be like if a mum is sitting out in the waiting room at the clinic you can just give it [whiteboard educational tool] to her to have a look at and then when she comes in we can talk about it as well. So it’s not too confrontational as well. She can already be thinking about some of those things before we talk.*

(Isobelle)

Mia explained that the existing oral health promotion resources needed to be complemented with oral health products *“Otherwise I think you end up just coming across a bit preachy*”:

*Health promotion from my experience is well delivered when it comes to the brochure and the paper source but you need to have something to back it up as a produce. So I believe a toothbrush and toothpaste pack already set up that when we give that information we hand that as well.*

(Mia)

#### 3.3.2. The Need for Policy Reform: “They’re Not Eligible for the Health Care Card”

One of the main recommendations made by the Aboriginal Health Workers focused on the gap in policy for some clients when referring to dental services. Some described the difficulty of accessing dental services through current referral pathways for some clients, particularly if the client did not meet the criteria for public or Aboriginal community-controlled dental services. The participants discussed that even though some clients had partners who had full-time work, dental treatment provided by private clinics would place financial stress on the family.

*With regards to the referral pathway it is a concern for adults from 18 and up who don’t have a Health Care Card because there are some families that the partner is working full-time, so therefore they’re not eligible for the health care card or pension card so where do they go from that because does it matter if you’ve got a full-time worker partner? Sometimes budget and finances are very tight, and that is a barrier that’s going to prevent an adult person to access a dentist if they can’t afford it. we do have our other referral pathways, like the Aboriginal. But then again, that’s a barrier for the Aboriginality [papers] as well and not all of our families will have that.*

(Mia)

While the Aboriginal Health Workers acknowledged that although the referral pathway implemented as part of the model of care was more culturally appropriate, there was still a clear gap for women who were not eligible:

*I guess it’s [referral pathway] a more culturally appropriate pathway. I’m still concerned that parents or mothers who don’t have access to concession cards won’t get free dental. I think everyone should get free dental.*

(Isobelle)

#### 3.3.3. Opportunities to Widen Reach: “I Just Want Everyone to Know About It”

When discussing the recommendations for the model of care, the Aboriginal Health Workers also identified the need to implement the model in other settings. The Aboriginal Health Workers described a couple of instances where the first point of contact for many Aboriginal and Torres Strait Islander women was in the hospital or through another service.

*I think Aboriginal health workers just even with other services, they do come across pregnant women. Sometimes they get them before we even do.*

(Kim)

*The hospital clinic definitely does [see pregnant women earlier] because that’s where all the referrals are coming from.*

(Isobelle)

The Aboriginal Health Workers spoke about how components of the model in other settings could complement their delivery of oral health promotion. Some comments identified that midwives providing a brochure at the initial antenatal visit could be helpful while other comments focused on the need to train the broader health workforce.

*We could also include it [brochure] in the cultural packs. So that way it’s still getting out there to those that don’t come to the program—they’re still going to get the information.*

(Rachel)

*I just want everyone to know about it [model of care]. I want you to go in and tell the Aboriginal midwife, to share it with everyone so everyone is aware of it. They might not have access to an Aboriginal health worker either. They still should still have this information.*

(Isobelle)

For clients who lived rurally, a few Aboriginal Health Workers in one workshop identified that Aboriginal health practitioners could play a role if oral health training was integrated into the curriculum. Kim, who had an Aboriginal Health Practitioner qualification, reflected on this:

*I think it’s true that you have a lot of Aboriginal health practitioners that work rurally. So maybe it could be something that could be put in with the training of the Aboriginal health practitioner. Because oral health is part of it, but you don’t touch on it. Nothing like this is for pregnant women.*

(Kim)

When asked about delivering the training in rural areas, Isobelle believed that it needed to be delivered face-to-face due to potential issues with internet connectivity.

*It [internet connectivity] just drops out all the time. So it’s not a good platform unless you’ve got a really good, secure network, and I’m not sure that they do. They [health workers in rural areas] might have out there, but they might not. But I would think—I prefer face-to-face.*

(Isobelle)

## 4. Discussion

This is the first time a culturally safe antenatal oral health model of care has been developed and piloted to train Aboriginal Health Workers to promote oral health among Aboriginal and Torres Strait Islander pregnant women worldwide [16]. This model of care included culturally appropriate resources, training, a screening tool, and a priority referral pathway. Although the impact of the COVID-19 pandemic limited the sample size, the findings revealed a high level of acceptability and satisfaction with the components of the model of care among the Aboriginal Health Workers, who also discussed how the model could be easily integrated into practice. These results were likely achieved because of the focus on PAR throughout this research [10], which was identified as a recommendation to create an oral health intervention among Aboriginal and Torres Strait Islander pregnant women in an earlier study [16]. As part of ethically conducted research across Canada and Australia, interventions that have involved capacity building Indigenous health staff in oral health undertake consultation and develop partnerships with key stakeholders and the community [34,35,36,37]. In this study, however, PAR was used purposefully so that Aboriginal Health Workers would be supported by the research team to direct the research and ensure that the actions and findings would meet the needs to promote oral health among Aboriginal and Torres Strait Islander pregnant women. PAR provided a framework where there was a high level of responsiveness to the needs of the Aboriginal Health Workers [16]. Using this approach is key to the translation of this model to other Aboriginal and Torres Strait Islander communities. PAR is also important to develop a sustainable model of oral healthcare for other pregnant women in communities where there may be an unequal power dynamic with the research team, such as with culturally and linguistically diverse communities or with refugee populations.

The pilot findings from the training workshop found individual improvements in oral health knowledge across all the Aboriginal Health Workers. Although the sample size was too small to determine whether this improvement was significant, there were large improvements in knowledge in several areas. These areas included misconceptions about oral health during pregnancy, the link between oral health and general health, eligibility to access public dental services, the safety of dental treatment during pregnancy, and early childhood oral health practices. These areas of knowledge are crucial to giving knowledge about the importance and safety of dental care during pregnancy, as well as advice about how to access dental services. In the international literature, both Indigenous and non-Indigenous pregnant women have misconceptions about oral health during pregnancy [6,38,39,40]. One review exploring the attitudes of Indigenous pregnant women found that some women avoided public dental services due to the complexity in navigating the service and suggested that experiences of racism may contribute to poor dental attendance [6]. The determinants of dental care use during pregnancy are multifactorial, encompassing demographic, socioeconomic, behavioural and psychological factors [41]. One of the psychological factors in this framework by Rocha, Arima, Werneck, Moysés, and Baldani [41] includes receiving oral health education, which suggests that this training could translate into enhancing access to dental care among Aboriginal and Torres Strait Islander pregnant women.

The Aboriginal Health Workers also reported a high level of confidence in discussing oral health care, screening pregnant women at risk of oral health problems and referring clients to the dental services after the training. The study by Smith et al. [42] similarly found that most Aboriginal Health Workers were confident to deliver dental advice to families about early childhood oral health following a training course. As Aboriginal and Torres Strait Islander women may also access other antenatal care providers such as midwives, other studies have also shown that training can improve confidence of antenatal care providers to deliver oral health promotion [18,43]. However, further research would be needed to determine the confidence of non-Indigenous antenatal care providers to deliver culturally safe oral health care following training.

The pilot findings suggest that the Grinnin’ Up Mums & Bubs training program provides Aboriginal Health Workers with the knowledge and confidence to promote oral health and undertake dental referrals for pregnant women. However, as identified in our findings, oral health can be a sensitive and challenging topic to discuss, particularly because poor oral health can be a source of shame and embarrassment for both non-Indigenous as well as Aboriginal and Torres Strait Islander peoples [10,44,45]. The Aboriginal Health Workers in this study discussed several strategies around initiating a conversation about oral health. The need to build rapport and trust has been identified in our earlier research [9,10]. However, this study highlights several other specific strategies including the need to be flexible, taking an indirect approach and using the oral health promotion resources to avoid a sense of shame, confrontation, and judgement. These strategies reflect a respectful and culturally safe practice where clients do not feel disempowered; rather, it enables Aboriginal and Torres Strait Islander women to engage in decision-making about their oral health during pregnancy [46].

The results from the Grinnin’ Up Mums & Bubs pilot suggests that the model could be translated into practice to improve the oral health outcomes of Aboriginal and Torres Strait Islander pregnant women. However, there is the need for further short and long-term evaluation with a larger sample. Follow up evaluation would need to assess whether the high oral health knowledge and confidence levels are sustained, and whether there is the support in place to ensure actual and long-term change in practice among the Aboriginal Health Workers. In addition, research should explore whether the model leads to an increase in accessing dental services during pregnancy and improves the oral health status and knowledge among Aboriginal and Torres Strait Islander women. These follow-up evaluations are important to measure to determine the long-term health and economic impact of the Grinnin’ Up Mums & Bubs model of care. Previous evaluations of the efficacy and cost-effectiveness of the MIOH model of care have established that it can reduce dental treatment costs in the long term [21], although more research is needed to determine the long-term impact of the Grinnin’ Up Mums & Bubs model.

In terms of long-term sustainability, particularly in the health service where the study was conducted, the existing antenatal service will need restructuring to integrate the Grinnin’ Up Mums & Bubs model. This integration will facilitate ongoing training as well as printing of resources and use of the dental referral pathways created. Additional strategies such as adding recommendations for discussing oral health and designating an Aboriginal Health Worker or Aboriginal health staff member to provide the training to new staff could also be included.

There is also the need to consider the policy barriers that impact on the effectiveness of the model. One of the main concerns identified in the pilot was that not all the Aboriginal Health Workers’ clients would be eligible to access the public dental services. Eligibility to access public dental services in NSW for adults are based on the client’s residency and whether they have a valid concession card [32]. Concession cards are available only if individuals fall under a relatively lower income bracket [47] or have a partial capacity to work [48]. Our earlier research confirms that the requirement for a concession card is a barrier for some Aboriginal and Torres Strait Islander pregnant women who may not be able to comfortably afford a dental check-up or treatment [9,10]. The provision of a free dental check-up or treatment during pregnancy, regardless of Health Care Card status, would be particularly important for clients of Aboriginal Health Workers who provide home-visiting antenatal support services to Aboriginal and Torres Strait Islander women [49,50]. Although the referral pathway to the public dental service was designed to reduce the institutional barrier in booking appointments, alternative solutions would need to be explored in other settings depending on the availability of affordable services. Opportunities should be explored to develop partnerships with local ACCHSs to allow for better coverage for patients who may not be ordinarily eligible to access public dental care. Currently in NSW, there is no avenue for subsidised care as it exists in other jurisdictions like Victoria and South Australia, which allows for targeted delivery of programs for vulnerable populations. University dental training programs could explore providing ineligible pregnant women with the appropriate assessments and treatments, although students would need additional training to ensure that their practice is culturally safe. Even without access to dental services, however, oral health advice can still provide a protective effect to a pregnant woman’s oral health. A recent study in Australia among pregnant women found that oral health knowledge delivered by midwives was effective in improving oral health even without a clear referral pathway in the short term [21]. As suggested in the findings, this advice could also be more effectively delivered among Aboriginal and Torres Strait Islander women if culturally appropriate oral health promotion resources are complemented with oral health products.

Lastly, it is important to consider the workload and time constraints experienced by Aboriginal Health Workers. A comprehensive review in Australia found that employee retention among Aboriginal Health Workers can be affected by stress due to the expectations and demands from both the workplace and community [51]. Thus, if there is staff turnover among the Aboriginal Health Workers, face-to-face workshops would need to be re-delivered to new staff. One approach that could increase dissemination of training may be to introduce an antenatal oral health module to the curricula of Aboriginal Health Workers as well as other health workers who deliver health care to Aboriginal and Torres Strait Islander pregnant women. As Aboriginal and Torres Strait Islander pregnant women may engage with different health care providers throughout the pregnancy, it is important that the broader antenatal health workforce are trained to promote oral health in ways that are culturally safe.

## 5. Conclusions

Grinnin’ Up Mums & Bubs is the first model of care that has been developed and piloted to be culturally safe to capacity build Aboriginal Health Workers to promote oral health among Aboriginal and Torres Strait Islander pregnant women. The various components of the model, including the resources, training, and referral pathway, were useful, culturally appropriate, and built on the existing knowledge and confidence of the Aboriginal Health Workers. The pilot’s findings also support a permanent integration of the model into the existing antenatal service. However, further evaluation is necessary to determine whether the model of care would be effective in changing practice among Aboriginal Health Workers and improving the oral health behaviours, practices and oral health status among Aboriginal and Torres Strait Islander pregnant women. The study also suggests the need for policy changes to ensure more Aboriginal and Torres Strait Islander women have access to affordable dental services during pregnancy as part of the model of care. Lastly, the study highlights that a one-size-fits-all solution would likely not be effective in promoting oral health among Aboriginal and Torres Strait Islander pregnant women. The PAR process in this study was essential to identifying needs as well as sustainable solutions for Aboriginal and Torres Strait Islander women in this community. For other populations in other settings, it is important that extensive engagement is undertaken to respond to the needs and voices of consumers, and to ensure program success and sustainability.

## Figures and Tables

**Table 1:** Categories and sub-categories.

Categories	Sub-Categories
Satisfaction with the model of care	Satisfaction with the training: “it built on my existing oral health knowledge” Satisfaction with the resources: “it’s a great visual prompt” Satisfaction with the referral pathway: “it’s a more culturally appropriate pathway”
Integration of the model in practice	Applying new knowledge in practice: “that information I can then put into practice” Renewed confidence to discuss oral health: “I feel more confident to answer questions”
Recommendations	Getting the message out there: “we’ve got to be flexible and meet the client’s needs” The need for policy reform: “they’re not eligible for the Health Care Card” Opportunities to widen reach: “I just want everyone to know about it”

**Table 2:** Likert agreement to post-training feedback statements.

Feedback Statements *	Mean Score (%)
The content was easy to understand	5.00 (100)
The material was relevant to my work	5.00 (100)
The training has given me knowledge to use when I give oral health advice	5.00 (100)
The screening tool is easy to use	5.00 (100)
The referral pathways would be appropriate to use	4.86 (97)
The length of the training was adequate	5.00 (100)
The training met the learning objectives	5.00 (100)
I would recommend this training to other Aboriginal Health Workers	5.00 (100)
I am satisfied with the quality of the presenters	5.00 (100)
I am satisfied with the quality of the PowerPoint	4.86 (97)
I am satisfied with the quality of the training manual	5.00 (100)
I am satisfied with the quality of the screening tool	5.00 (100)
I am satisfied with the quality of the supporting oral health resources (brochure, whiteboard resource, magnet)	5.00 (100)
The training presentation was culturally appropriate	4.86 (97)
The training manual was culturally appropriate	5.00 (100)
The screening tool was culturally appropriate	5.00 (100)
The referral pathways were culturally appropriate	4.86 (97)
The supporting oral health resources (brochure, whiteboard resource, magnet) was culturally appropriate	5.00 (100)

* Statements rated using a numerical (1–5) Likert scale where 1 = strongly disagree and 5 = strongly agree.

**Table 3:** Change in oral health knowledge pre–post training.

Section	Knowledge Question	Knowledge Scores		
		Baseline (%)	Post (%)	Change Pre-Post (%)
Section 1: Oral health changes and misconceptions during pregnancy	K1. Pregnant women are at more risk of getting oral health problems compared to other women	4 (57.1)	6 (85.7)	29% *
	K2. Gums can become red and swollen during pregnancy	5 (71.4)	6 (85.7)	14%
	K3. Women should brush teeth after vomiting to avoid wearing down teeth	2 (28.6)	7 (100)	71% *
	K4. The baby draws calcium out of the mother’s teeth during pregnancy	1 (14.3)	7 (100)	86% *
Section 2: Link between maternal oral health and pregnancy outcomes	K5. Severe gum disease has been linked to: late delivery of babies	3 (42.9)	5 (71.4)	29% *
	K6. Severe gum disease has been linked to: low birth weight of babies	2 (28.6)	4 (57.1)	29% *
	K7. Severe gum disease has been linked to: pregnancy loss	2 (28.6)	3 (42.9)	14%
Section 4: Antenatal care guidelines and recommendations on the role of Aboriginal Health Workers	K8. Based on current pregnancy care guidelines, all antenatal care providers (including AHWs) should: give oral health advice to pregnant women	5 (71.4)	7 (100)	29% *
	K9. Based on current pregnancy care guidelines, all antenatal care providers (including AHWs) should: avoid asking pregnant women questions about potential dental problems	6 (85.7)	7 (100)	14%
	K10. Based on current pregnancy care guidelines, all antenatal care providers (including AHWs) should: avoid recommending women to see dental services	6 (85.7)	7 (100)	14%
	K11. Based on current pregnancy care guidelines, all antenatal care providers (including AHWs) should: recommend that every child should see a dentist by the age of one	6 (85.7)	6 (85.7)	0%
Section 5: Accessing public dental services	K12. Only adults with Health Care Cards/Pension Cards can access public dental services	5 (71.4)	7 (100)	29% *
	K13. Pregnant women, who are eligible to go to public dental services, will have to wait longer than 3 months for an appointment	1 (14.3)	7 (100)	86% *
	K14. Not all children (under 18 years) can access public dental services	4 (57.1)	7 (100)	43% *
	K15. Some children can receive the Child Benefits Schedule (AUD 1000) from Medicare to go to a private dentist	6 (85.7)	6 (85.7)	0%
Section 6: Safety of specific dental treatments during pregnancy	K16. All antibiotics are safe during pregnancy	2 (28.6)	4 (57.1)	29% *
	K17. All pain relief medicines are safe during pregnancy	4 (57.1)	4 (57.1)	0%
	K18. Most dental treatments are safe during pregnancy	4 (57.1)	6 (85.7)	29% *
	K19. Dental X-rays are safe during pregnancy	3 (42.9)	5 (71.4)	29% *
	K20. Local anaesthesia (numbing injections) are safe during pregnancy	2 (28.6)	5 (71.4)	43% *
Section 7: Early childhood oral health practices	K21. Putting a baby to bed with a bottle increases the baby’s risk of dental decay	7 (100)	7 (100)	0%
	K22. Adding fruit juice to the baby’s bottle will not increase the baby’s risk of dental decay as long as there are “no added sugars”	6 (85.7)	7 (100)	14%
	K23. Adding honey (or another sugar sweetener) to the baby’s dummy will not increase the baby’s risk of dental decay	6 (85.7)	6 (85.7)	0%
	K24. Sharing a spoon with the baby will not increase the baby’s risk of dental decay	5 (71.4)	7 (100)	29% *

* >25% improvement in knowledge.

**Table 4:** Change in oral health confidence pre-post training.

Confidence Statement	Knowledge Scores		
	Baseline (%)	Post (%)	Change Pre-Post (%)
1. How confident are you in discussing good oral health care with a woman during her pregnancy?	3.57 (71)	4.43 (89)	17%
2. How confident are you in asking screening questions to identify if a pregnant woman needs to see the dentist?	3.86 (77)	4.86 (97)	20%
3. How confident are you in referring clients at risk of poor oral health to dental services?	4.43 (89)	5 (100)	11%

## Data Availability

The data presented in this study are available upon reasonable request from the corresponding author.

## Supplementary Materials

The following are available online at https://www.mdpi.com/article/10.3390/ijerph18189576/s1. Images for the oral health promotion resources can be located in Supplmentary File S1. The pre- and post-training questionnaires can be located in Supplementary File S2.

## References

[1] George A., Johnson M., Blinkhorn A., Ellis S., Bhole S., Ajwani S. (2010). Promoting oral health during pregnancy: Current evidence and implications for Australian midwives. J. Clin. Nurs..

[2] Castilho A.R.F.D., Mialhe F.L., Barbosa T.D.S., Puppin-Rontani R.M. (2013). Influence of family environment on children’s oral health: A systematic review. J. Pediatr..

[3] Dye B.A., Vargas C.M., Lee J.J., Magder L., Tinanoff N. (2011). Assessing the Relationship Between Children’s Oral Health Status and That of Their Mothers. J. Am. Dent. Assoc..

[4] Daalderop L.A., Wieland B.V., Tomsin K., Reyes L., Kramer B.W., Vanterpool S.F., Been J.V. (2018). Periodontal Disease and Pregnancy Outcomes: Overview of Systematic Reviews. JDR Clin. Trans. Res..

[5] George A., Johnson M., Blinkhorn A., Ajwani S., Bhole S., Yeo A., Ellis S. (2013). The oral health status, practices and knowledge of pregnant women in south-western Sydney. Aust. Dent. J..

[6] Kong A.C., Ramjan L., Sousa M.S., Gwynne K., Goulding J., Jones N., Srinivas R., Rambaldini B., Moir R., George A. (2019). The oral health of Indigenous pregnant women: A mixed-methods systematic review. Women Birth.

[7] Australian Indigenous Health InfoNet (2020). Overview of Aboriginal and Torres Strait Islander Health Status. https://healthinfonet.ecu.edu.au/learn/health-facts/overview-aboriginal-torres-strait-islander-health-status.

[8] Durey A., Bessarab D., Slack-Smith L. (2016). The mouth as a site of structural inequalities; the experience of Aboriginal Australians. Community Dent. Health.

[9] Kong A., Dickson M., Ramjan L., Sousa M.S., Goulding J., Chao J., George A. (2021). A Qualitative Study Exploring the Experiences and Perspectives of Australian Aboriginal Women on Oral Health during Pregnancy. Int. J. Environ. Res. Public Health.

[10] Kong A.C., Sousa M.S., Ramjan L., Dickson M., Goulding J., Gwynne K., Talbot F., Jones N., Srinivas R., George A. (2020). “Got to build that trust”: The perspectives and experiences of Aboriginal health staff on maternal oral health. Int. J. Equity Health.

[11] Australian Institute of Aboriginal and Torres Strait Islander Studies Proof of Aboriginality. https://aiatsis.gov.au/family-history/you-start/proof-aboriginality.

[12] Herring S., Spangaro J., Lauw M., McNamara L. (2013). The intersection of trauma, racism, and cultural competence in effective work with Aboriginal people: Waiting for trust. Aust. Soc. Work..

[13] Australian Institute of Aboriginal and Torres Strait Islander Studies Understanding the Challenges. https://aiatsis.gov.au/family-history/you-start/understanding-challenges.

[14] Australian Institute of Aboriginal and Torres Strait Islander Studies Indigenous Names. https://aiatsis.gov.au/family-history/you-start/indigenous-names.

[15] Australian Institute of Aboriginal and Torres Strait Islander Studies Thinking about Place. https://aiatsis.gov.au/family-history/you-start/thinking-about-place.

[16] Villarosa A.C., Villarosa A.R., Salamonson Y., Ramjan L.M., Sousa M.S., Srinivas R., Jones N., George A. (2018). The role of indigenous health workers in promoting oral health during pregnancy: A scoping review. BMC Public Health.

[17] George A., Dahlen H.G., Blinkhorn A., Ajwani S., Bhole S., Ellis S., Yeo A., Elcombe E., Johnson M. (2018). Evaluation of a midwifery initiated oral health-dental service program to improve oral health and birth outcomes for pregnant women: A multi-centre randomised controlled trial. Int. J. Nurs. Stud..

[18] George A., Lang G., Johnson M., Ridge A., de Silva A.M., Ajwani S., Bhole S., Blinkhorn A., Dahlen H.G., Ellis S. (2016). The evaluation of an oral health education program for midwives in Australia. Women Birth.

[19] Dahlen H.G., Johnson M., Hoolsema J., Norrie T.P., Ajwani S., Blinkhorn A., Bhole S., Ellis S., Srinivas R., Yaacoub A. (2019). Process evaluation of the midwifery initiated oral health-dental service program: Perceptions of midwives in Greater Western Sydney, Australia. Women Birth.

[20] George A., Villarosa A.R., Patterson Norrie T., Hoolsema J., Dahlen H.G., Ajwani S., Bhole S., Blinkhorn A., Srinivas R., Yaacoub A. (2019). Process evaluation of the midwifery initiated oral health-dental service program: Perceptions of pregnant women. Aust. Dent. J..

[21] Tannous K., George A., Ahmed M.U., Blinkhorn A., Dahlen H.G., Skinner J., Ajwani S., Bhole S., Yaacoub A., Srinivas R. (2021). Economic evaluation of the Midwifery-Initiated Oral Health Dental Service program in Australia. BMJ Open.

[22] World Health Organization Publication Consultation on Implementation Guidance: How to Integrate Non-Communicable Diseases with National HIV/AIDS, TB, Sexual and Reproductive Health Programmes, and into the Health System. https://www.who.int/news-room/articles-detail/publication-consultation-on-implementation-guidance-how-to-integrate-non-communicable-diseases-with-national-hiv-aids-tb-sexual-and-reproductive-health-programmes-and-into-the-health-system.

[23] Sivertsen N., Anikeeva O., Deverix J., Grant J. (2020). Aboriginal and Torres Strait Islander family access to continuity of health care services in the first 1000 days of life: A systematic review of the literature. BMC Health Serv. Res..

[24] Baum F., MacDougall C., Smith D. (2006). Participatory action research. J. Epidemiol. Community Health.

[25] Ozanne J.L., Saatcioglu B. (2008). Participatory Action Research. J. Consum. Res..

[26] Kovach M. (2010). Indigenous Methodologies: Characteristics, Conversations, and Contexts.

[27] Bessarab D., Ng’andu B. (2010). Yarning about yarning as a legitimate method in Indigenous research. Int. J. Crit. Indig. Stud..

[28] George A., Dahlen H.G., Blinkhorn A., Ajwani S., Bhole S., Ellis S., Yeo A., Elcombe E., Sadozai A., Johnson M. (2016). Measuring oral health during pregnancy: Sensitivity and specificity of a maternal oral screening (MOS) tool. BMC Pregnancy Childbirth.

[29] Centre for Oral Health Strategy (2017). Priority Oral Health Program (POHP) and Waiting List Management.

[30] Silk H., Douglass A.B., Douglass J.M., Silk L. (2008). Oral health during pregnancy. Am. Fam. Physician.

[31] Russell S.L., Mayberry L.J. (2008). Pregnancy and Oral Health: A Review and Recommendations to Reduce Gaps in Practice and Research. MCN Am. J. Matern. Child Nurs..

[32] Centre for Oral Health Strategy Eligibility of Persons for Public Oral Health Care in NSW. https://www1.health.nsw.gov.au/pds/ActivePDSDocuments/PD2017_027.pdf.

[33] Hsieh H.-F., Shannon S.E. (2005). Three Approaches to Qualitative Content Analysis. Qual. Health Res..

[34] Slade G.D., Bailie R.S., Roberts-Thomson K., Leach A.J., Raye I., Endean C., Simmons B., Morris P. (2011). Effect of health promotion and fluoride varnish on dental caries among Australian Aboriginal children: Results from a community-randomized controlled trial. Community Dent. Oral Epidemiol..

[35] Blinkhorn F., Brown N., Freeman R., Humphris G., Martin A., Blinkhorn A. (2012). A phase II clinical trial of a dental health education program delivered by aboriginal health workers to prevent early childhood caries. BMC Public Health.

[36] Pacza T., Steele L., Tennant M. (2001). Development of oral health training for rural and remote Aboriginal health workers. Aust. J. Rural Health.

[37] First Nations Health Authority Healthy Smiles for Life. https://www.fnha.ca/WellnessSite/WellnessDocuments/FNHA_HealthySmilesforLife_OralHealthStrategy2014.pdf.

[38] Gonik B., Wilson E., Mayberry M., Joarder B.Y. (2017). Pregnant Patient Knowledge and Behavior Regarding Perinatal Oral Health. Am. J. Perinatol..

[39] Detman L.A., Cottrell B.H., Denis-Luque M.F. (2010). Exploring Dental Care Misconceptions and Barriers in Pregnancy. Birth.

[40] Saddki N., Yusoff A., Hwang Y.L. (2010). Factors associated with dental visit and barriers to utilisation of oral health care services in a sample of antenatal mothers in Hospital Universiti Sains Malaysia. BMC Public Health.

[41] Rocha J.S., Arima L.Y., Werneck R.I., Moysés S.J., Baldani M.H. (2018). Determinants of Dental Care Attendance during Pregnancy: A Systematic Review. Caries Res..

[42] Smith L., Blinkhorn F., Moir R., Brown N., Blinkhorn A. (2016). User assessment of an early childhood oral health education training course for Aboriginal Health Workers. Int. J. Health Promot. Educ..

[43] Lim M., Riggs E., Shankumar R., Marwaha P., Kilpatrick N. (2018). Midwives’ and women’s views on accessing dental care during pregnancy: An Australian qualitative study. Aust. Dent. J..

[44] George A., Johnson M., Duff M., Blinkhorn A., Ajwani S., Bhole S., Ellis S. (2011). Maintaining oral health during pregnancy: Perceptions of midwives in Southwest Sydney. Collegian.

[45] Jamieson L.M., Parker E.J., Richards L. (2008). Using qualitative methodology to inform an Indigenous-owned oral health promotion initiative in Australia. Health Promot. Int..

[46] Curtis E., Jones R., Tipene-Leach D., Walker C., Loring B., Paine S.-J., Reid P. (2019). Why cultural safety rather than cultural competency is required to achieve health equity: A literature review and recommended definition. Int. J. Equity Health.

[47] Services Australia Health Care Card: Who Can Get a Card. https://www.servicesaustralia.gov.au/individuals/services/centrelink/health-care-card.

[48] Services Australia Pensioner Concession Card: Who Can Get a Card. https://www.servicesaustralia.gov.au/individuals/services/centrelink/pensioner-concession-card/who-can-get-card.

[49] Best E. (2011). Closing the gap through innovative maternity care (The Aboriginal Maternal and Infant Health Service). Women Birth.

[50] McDonald M., Moore T.G., Goldfeld S. (2012). Sustained Home Visiting for Vulnerable Families and Children: A Literature Review of Effective Programs.

[51] Deroy S., Schütze H. (2019). Factors supporting retention of Aboriginal health and wellbeing staff in Aboriginal health services: A comprehensive review of the literature. Int. J. Equity Health.

