# Supplementary material for: Aboriginal Health Workers Promoting Oral Health among Aboriginal and Torres Strait Islander Women during Pregnancy: Development and Pilot Testing of the Grinnin’ Up Mums & Bubs Program
Source: Int J Environ Res Public Health. 2021 Sep 11;18(18):9576. doi: 10.3390/ijerph18189576 (PMC8471975; doi:10.3390/ijerph18189576)

Supplementary File S1. Images of the Grinnin' Up Mums & Bubs oral health promotion resources

Oral health educational brochure: Side A

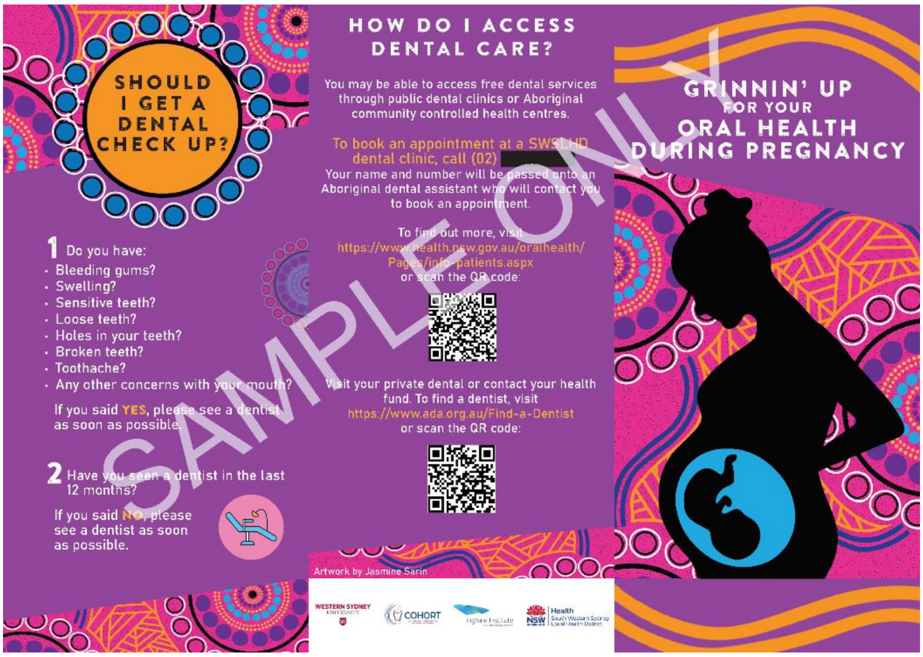

Oral health educational brochure: Side B

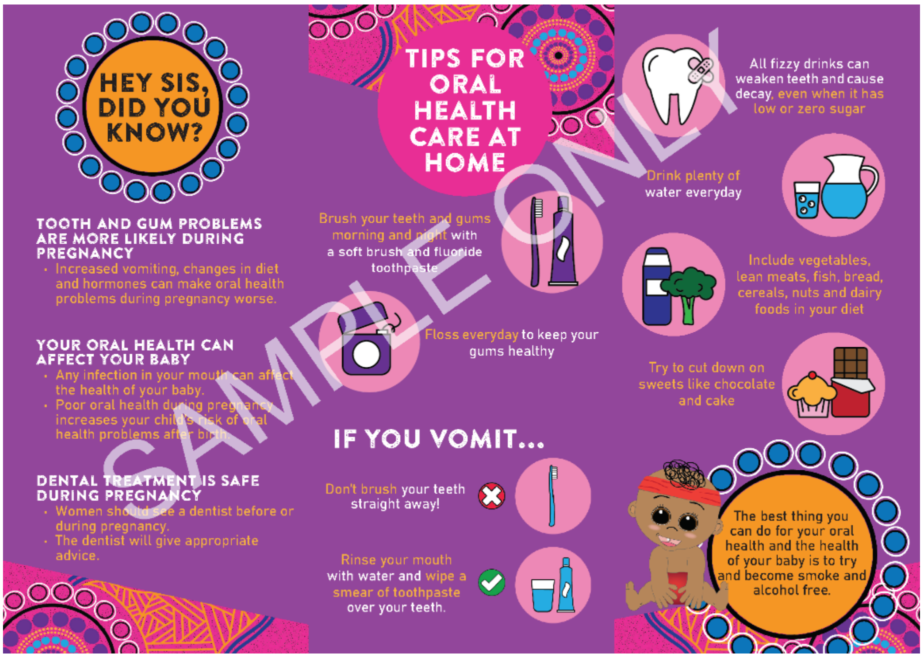

Fridge magnet (larger size)

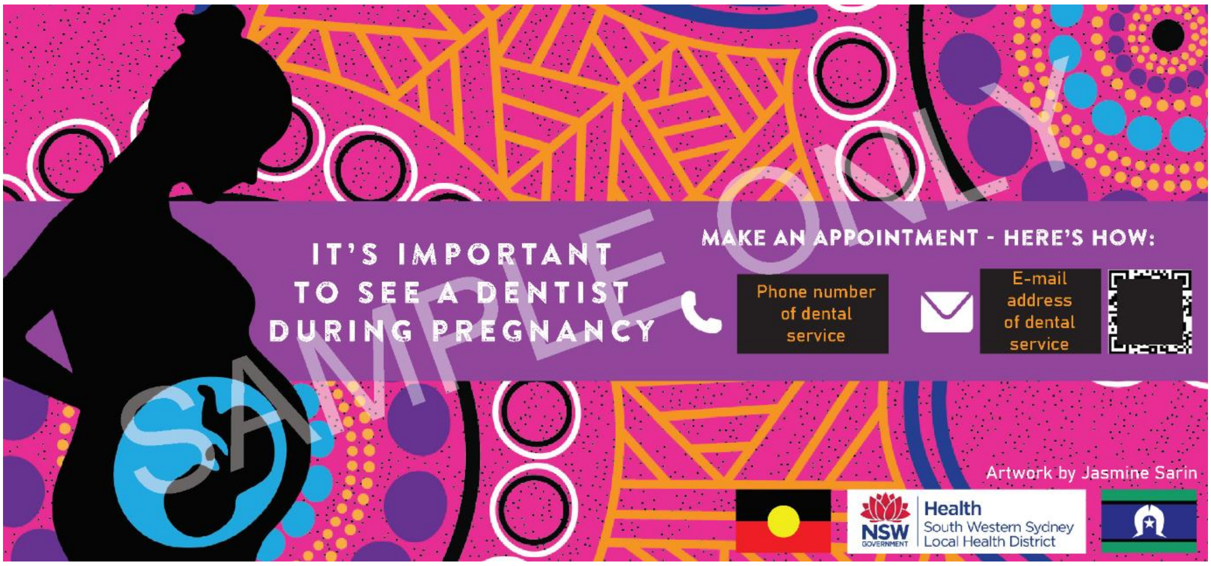

Fridge magnet (smaller size)

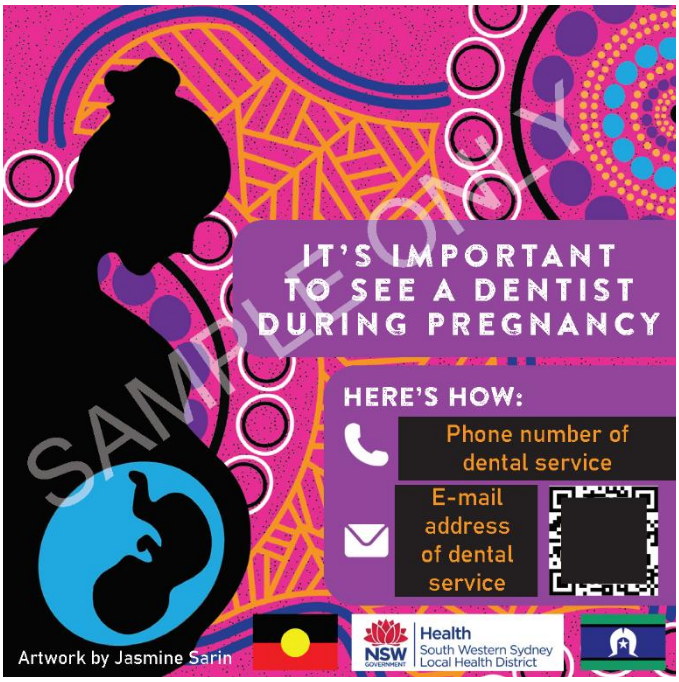

Whiteboard educational tool: Side A

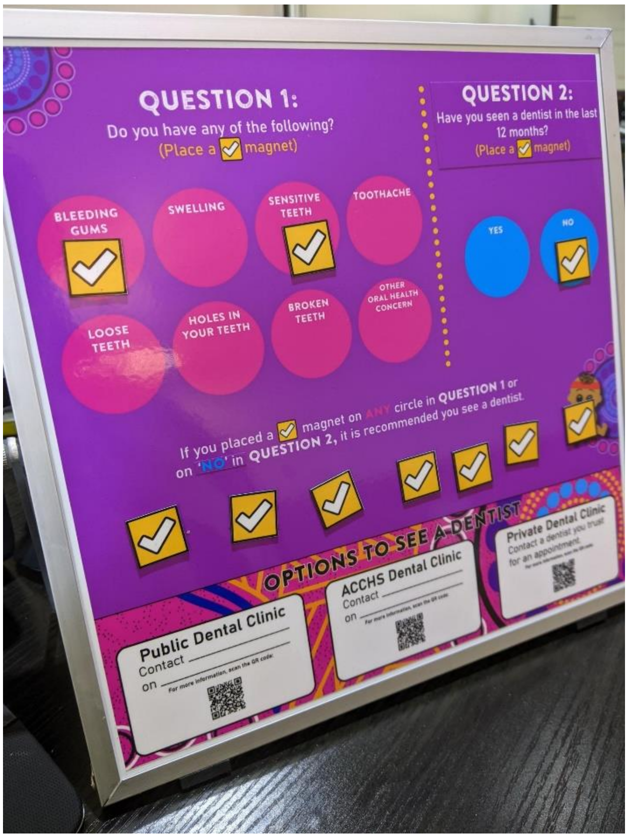

Whiteboard educational tool: Side B

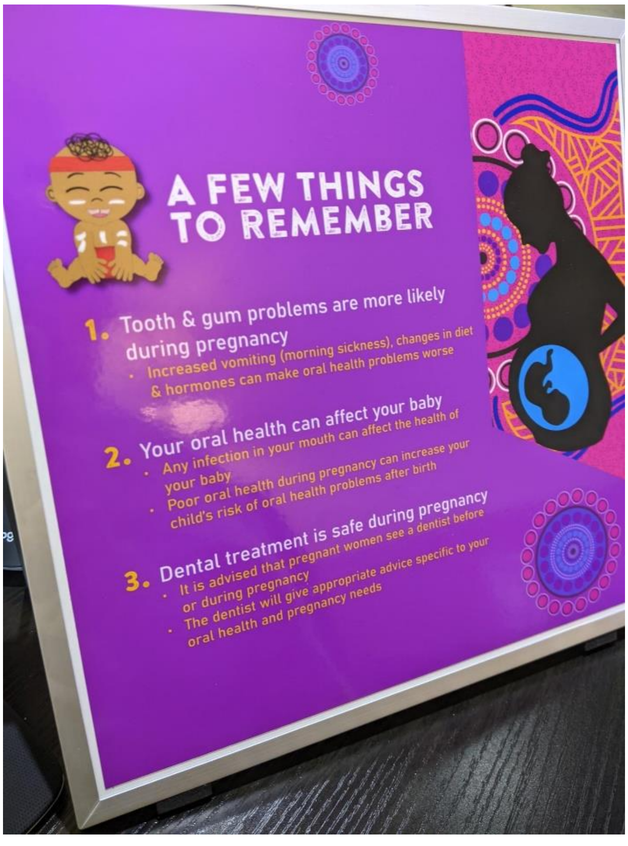

Grinnin' Up Bubs & Bubs training workbook

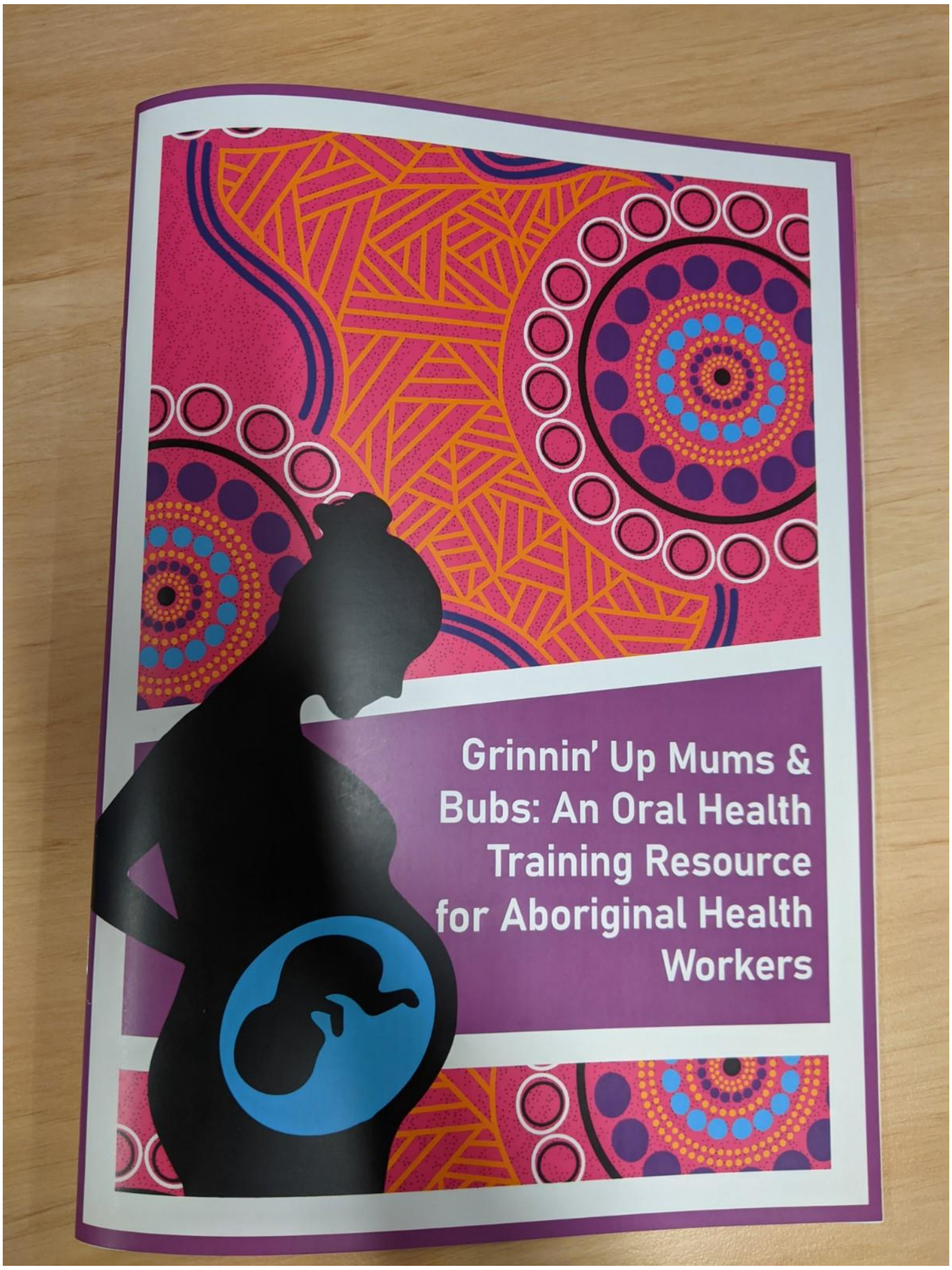

Supplement: Supplementary file 1 [file ijerph-18-09576-s001.zip › SupplementaryFileS1_OralHealthPromotionResources.pdf]
