# Supplementary material for: Aboriginal Health Workers Promoting Oral Health among Aboriginal and Torres Strait Islander Women during Pregnancy: Development and Pilot Testing of the Grinnin’ Up Mums & Bubs Program
Source: Int J Environ Res Public Health. 2021 Sep 11;18(18):9576. doi: 10.3390/ijerph18189576 (PMC8471975; doi:10.3390/ijerph18189576)
Supplement: Supplementary file 1 [file ijerph-18-09576-s001.zip › SupplementaryFileS2_PrePostTrainingQuestionnaire.pdf]

Supplementary File S2. Pre and Post-training questionnaires

Baseline (Pre-Training) Survey

Your unique ID (to link pre and post surveys):
Last 3 digits of your mobile number): _ _ _
Date of Birth (DD/MM/YYYY): _ _ / _ _ / _ _ _ _

Grinnin' Up Mums & Bubs

The following questions explore your knowledge and confidence about oral health.

Before you complete this survey, please make sure that you have read the information sheet and have provided consent. Please answer all questions. Most questions require you to select a box to indicate your answer. If you are not sure what the answer is, please select 'Don't know' as we want to know how effective our training program will be in improving your knowledge.

Section S1 – True/False Knowledge Questions

The following questions will explore your knowledge about 1) oral health during pregnancy, 2) current oral health guidelines, 3) accessing dental services, 4) safety of dental treatment and 5) oral health after the baby is born.

Please tick 'True' or 'False' or 'Don't know' for the following questions to the best of your knowledge.

		True	False	Don't Know
**1.**	The following statements are about **oral health during pregnancy**:			
	Pregnant women are at more risk of getting oral health problems compared to other women.	☐	☐	☐
	Gums can become red and swollen during pregnancy.	☐	☐	☐
	Women should brush teeth after vomiting to avoid wearing down teeth.	☐	☐	☐
	The baby draws calcium out of the mother's teeth during pregnancy.	☐	☐	☐
	Severe gum disease has been linked to:			
	Late delivery of babies	☐	☐	☐
	Low birth weight of babies	☐	☐	☐
	Pregnancy loss	☐	☐	☐

**Please tick 'True' or 'False' or 'Don't know' for the following questions to the best of your knowledge.**				
		**True**	**False**	**Don't Know**
**2.**	**Based on current pregnancy care guidelines**, all antenatal care providers (including Aboriginal Health Workers) should:			
	Give oral health advice to pregnant women	☐	☐	☐
	Avoid asking pregnant women questions about potential dental problems	☐	☐	☐
	Avoid recommending women to see dental services	☐	☐	☐
	Recommend that every child should see a dentist by the age of one	☐	☐	☐
**3.**	The following statements relate to **accessing the dentist.**			
	Only adults with Health Care Cards/Pension Cards can access public dental services.	☐	☐	☐
	Pregnant women, who are eligible to go to public dental services, will have to wait longer than 3 months for an appointment.	☐	☐	☐
	Not all children (under 18 years) can access public dental services.	☐	☐	☐
	Some children can receive the Child Benefits Schedule ($1000) from Medicare to go to a private dentist.	☐	☐	☐
**4.**	The following are **safe** during pregnancy:			
	All antibiotics	☐	☐	☐
	All pain relief medicine	☐	☐	☐
	Most dental treatments	☐	☐	☐
	Dental x-rays	☐	☐	☐
	Local anaesthesia (numbing injections)	☐	☐	☐

Please tick 'True' or 'False' or 'Don't know' for the following questions to the best of your knowledge.				
		True	False	Don't Know
5.	The following statements are about **oral health after the baby is born**.			
	Putting a baby to bed with a bottle increases the baby's risk of dental decay.	☐	☐	☐
	Adding fruit juice to the baby's bottle won't increase the baby's risk of dental decay as long as there are 'no added sugars'.	☐	☐	☐
	Adding honey (or another sugar sweetener) to the baby's dummy won't increase the baby's risk of dental decay.	☐	☐	☐
	Sharing a spoon with the baby will not increase the baby's risk of dental decay.	☐	☐	☐

Section S2 - Level of Confidence

How confident are you in discussing good oral health care with a woman during her pregnancy?

Not confident at all				Completely confident
☐	☐	☐	☐	☐
1	2	3	4	5

How confident are you in asking screening questions to identify if a pregnant woman needs to see the dentist?

Not confident at all				Completely confident
☐	☐	☐	☐	☐
1	2	3	4	5

How confident are you in referring clients at risk of poor oral health to dental services?

Not confident at all				Completely confident
☐	☐	☐	☐	☐
1	2	3	4	5

Section S3 - General Information About Yourself

1. What is your highest level of education?

- ☐ Primary school
- ☐ High school
- ☐ TAFE/Diploma
- ☐ Bachelor degree
- ☐ Honours
- ☐ Graduate certificate/graduate diploma
- ☐ Masters
- ☐ PhD
- ☐ Other (*Please specify e.g. if you are currently studying*)

  ____

2. How long (in years) have you been working as an Aboriginal Health Worker? *(If less than 1 year, please advise how many months)*

________________________________ years / months *(please circle)*

3. Have you previously received any education or training regarding oral health care for pregnant women <u>in any previous or current role</u>?

- ☐ Yes
- ☐ No

If yes, where did you receive this education or training?

____

Thank you very much for your time

Post-Training Survey

Your unique ID (to link pre and post surveys):
Last 3 digits of your mobile number): _ _ _
Date of Birth (DD/MM/YYYY): _ _ / _ _ / _ _ _ _

Grinnin' Up Mums & Bubs

The following questions explore your knowledge and confidence about oral health.

Before you complete this survey, please make sure that you have read the information sheet and have provided consent. Please answer all questions. Most questions require you to select a box to indicate your answer. If you are not sure what the answer is, please select 'Don't know' as we want to know how effective our training program will be in improving your knowledge.

Section S1 – True/False Knowledge Questions

The following questions will explore your knowledge about 1) oral health during pregnancy,
2) current oral health guidelines, 3) accessing dental services, 4) safety of dental treatment and 5) oral health after the baby is born.

Please tick 'True' or 'False' or 'Don't know' for the following questions to the best of your knowledge.

		True	False	Don't Know
**1.**	The following statements are about **oral health during pregnancy**:			
	Pregnant women are at more risk of getting oral health problems compared to other women.	☐	☐	☐
	Gums can become red and swollen during pregnancy.	☐	☐	☐
	Women should brush teeth after vomiting to avoid wearing down teeth.	☐	☐	☐
	The baby draws calcium out of the mother's teeth during pregnancy.	☐	☐	☐
	Severe gum disease has been linked to:			
	Late delivery of babies	☐	☐	☐
	Low birth weight of babies	☐	☐	☐
	Pregnancy loss	☐	☐	☐

**Please tick 'True' or 'False' or 'Don't know' for the following questions to the best of your knowledge.**				
		**True**	**False**	**Don't Know**
**2.**	**Based on current pregnancy care guidelines**, all antenatal care providers (including Aboriginal Health Workers) should:			
	Give oral health advice to pregnant women	☐	☐	☐
	Avoid asking pregnant women questions about potential dental problems	☐	☐	☐
	Avoid recommending women to see dental services	☐	☐	☐
	Recommend that every child should see a dentist by the age of one	☐	☐	☐
**3.**	The following statements relate to **accessing the dentist.**			
	Only adults with Health Care Cards/Pension Cards can access public dental services.	☐	☐	☐
	Pregnant women, who are eligible to go to public dental services, will have to wait longer than 3 months for an appointment.	☐	☐	☐
	Not all children (under 18 years) can access public dental services.	☐	☐	☐
	Some children can receive the Child Benefits Schedule ($1000) from Medicare to go to a private dentist.	☐	☐	☐
**4.**	The following are **safe** during pregnancy:			
	All antibiotics	☐	☐	☐
	All pain relief medicine	☐	☐	☐
	Most dental treatments	☐	☐	☐
	Dental x-rays	☐	☐	☐
	Local anaesthesia (numbing injections)	☐	☐	☐

**Please tick 'True' or 'False' or 'Don't know' for the following questions to the best of your knowledge.**				
		**True**	**False**	**Don't Know**
**5.**	The following statements are about **oral health after the baby is born**.			
	Putting a baby to bed with a bottle increases the baby's risk of dental decay.	☐	☐	☐
	Adding fruit juice to the baby's bottle won't increase the baby's risk of dental decay as long as there are 'no added sugars'.	☐	☐	☐
	Adding honey (or another sugar sweetener) to the baby's dummy won't increase the baby's risk of dental decay.	☐	☐	☐
	Sharing a spoon with the baby will not increase the baby's risk of dental decay.	☐	☐	☐

Section S2 - Level of Confidence

How confident are you in discussing good oral health care with a woman during her pregnancy?

Not confident at all — Completely confident

☐ 1 — ☐ 2 — ☐ 3 — ☐ 4 — ☐ 5

How confident are you in asking screening questions to identify if a pregnant woman needs to see the dentist?

Not confident at all — Completely confident

☐ 1 — ☐ 2 — ☐ 3 — ☐ 4 — ☐ 5

How confident are you in referring clients at risk of poor oral health to dental services?

Not confident at all — Completely confident

☐ 1 — ☐ 2 — ☐ 3 — ☐ 4 — ☐ 5

Section S3 - Feedback

For each statement below, please choose a number that best represents how you felt after the training (1 = strongly disagree, 7 = strongly agree)

		Strongly disagree ↓				Strongly agree ↓.
**1. When it comes to the <u>training content</u>:**						
**a.**	The content was easy to understand	1	2	3	4	5
**b.**	The material was relevant to my work	1	2	3	4	5
**c.**	The training has given me knowledge to use when I give oral health advice	1	2	3	4	5
**d.**	The screening tool is easy to use	1	2	3	4	5
**e.**	The referral pathways would be appropriate to use	1	2	3	4	5
**f.**	The length of the training was adequate	1	2	3	4	5
**g.**	The training met the learning objectives	1	2	3	4	5
**h.**	I would recommend this training to other Aboriginal Health Workers	1	2	3	4	5
**2. I am satisfied with the quality of the:**		Strongly disagree ↓				Strongly agree ↓.
**a.**	Presenters	1	2	3	4	5
**b.**	PowerPoint	1	2	3	4	5
**c.**	Training manual	1	2	3	4	5
**d.**	Screening tool	1	2	3	4	5
**e.**	Supporting oral health resources (brochure, whiteboard resource, magnet)	1	2	3	4	5
**3. The following items were <u>culturally appropriate</u>:**		Strongly disagree ↓				Strongly agree ↓.
**a.**	Training presentation	1	2	3	4	5
**b.**	Training manual	1	2	3	4	5
**c.**	Screening tool	1	2	3	4	5
**d.**	Referral pathways	1	2	3	4	5
**e.**	Supporting oral health resources (brochure, whiteboard resource, magnet)	1	2	3	4	5

4. What did you like about the training?
5. What didn't you like about the training?
6. Do you have any recommendations to improve the program?

Thank you very much for your time
